# Integrating Phylodynamics and Epidemiology to Estimate Transmission Diversity in Viral Epidemics

**DOI:** 10.1371/journal.pcbi.1002876

**Published:** 2013-01-31

**Authors:** Gkikas Magiorkinis, Vana Sypsa, Emmanouil Magiorkinis, Dimitrios Paraskevis, Antigoni Katsoulidou, Robert Belshaw, Christophe Fraser, Oliver George Pybus, Angelos Hatzakis

**Affiliations:** 1 Department of Hygiene, Epidemiology and Medical Statistics, Medical School, University of Athens, Athens, Greece; 2 Department of Zoology, University of Oxford, Oxford, United Kingdom; 3 School of Public Health, Imperial College, London, United Kingdom; University of California San Diego, United States of America

## Abstract

The epidemiology of chronic viral infections, such as those caused by Hepatitis C Virus (HCV) and Human Immunodeficiency Virus (HIV), is affected by the risk group structure of the infected population. Risk groups are defined by each of their members having acquired infection through a specific behavior. However, risk group definitions say little about the transmission potential of each infected individual. Variation in the number of secondary infections is extremely difficult to estimate for HCV and HIV but crucial in the design of efficient control interventions. Here we describe a novel method that combines epidemiological and population genetic approaches to estimate the variation in transmissibility of rapidly-evolving viral epidemics. We evaluate this method using a nationwide HCV epidemic and for the first time co-estimate viral generation times and superspreading events from a combination of molecular and epidemiological data. We anticipate that this integrated approach will form the basis of powerful tools for describing the transmission dynamics of chronic viral diseases, and for evaluating control strategies directed against them.

## Author Summary

To design strategies that efficiently mitigate an epidemic requires estimates of how many people each carrier is likely to infect, what is the variation of this number among infections, and what is the time needed for these transmissions to take place. The disciplines of epidemiology and population genetics independently provide partial answers to these questions by analysing surveillance data and molecular sequences, respectively. Here we propose a novel integration of the two fields that can reveal the underlying transmission dynamics of rapidly-evolving viruses such as HIV or HCV. We explore a well-described nationwide HCV epidemic and show that our method provides new insights into the nature and variation of HCV transmission among infected individuals. We suggest that this approach could form the basis of new tools that can help in the design of effective public health interventions targeting the spread of viral pathogens.

## Introduction

Mathematical epidemiology describes the spread of infectious diseases and aims to aid in the design of effective public health interventions [1]–[3]. Central to this endeavour is the basic reproductive number (*R*_0_) of an infectious disease, the mean number of secondary infections per primary infection in a completely susceptible population [4] (for notations see Table 1). Under simple epidemiological scenarios, in which all infected individuals behave identically, *R*_0_ depends on the transmission probability per contact with a susceptible individual, the duration of infectiousness and the rate at which new contacts are made [2], [4], [5]. However, studies on sexually transmitted and vector-borne infections indicate that infected individuals behave far from identically and that variation in the number of secondary infections per infected individual can play a major role in epidemic dynamics. For example, some researchers have invoked the so-called 20–80 rule to describe the finding that approximately 20% of infected individuals are responsible for 80% of onward transmission [3], [6], [7]. The term ‘superspreaders’ has been coined to describe hosts that contribute disproportionately to onward infection.

**Table 1:** Abbreviations and terms used throughout the manuscript.

Symbol	Name	Statistical definiton	Units
*R*_0_	Basic reproductive number or ratio	Mean number of secondary infections	Number of infections
*R*_0,a_	Basic reproductive number or ratio of the transmitter group assuming a transmitter, non-transmitter secondary infections model	Mean number of secondary infections	Number of infections
*Z*	Number of secondary infections per infected individual	Random variable	Number of infections
*Z*_a_	Number of secondary infections of the transmitter group assuming a transmitter, non-transmitter secondary infections model	Random variable	Number of infections
*N*	Number of prevalent cases	-	Number of infected people
*N_e_*	Effective number of infections	-	Number of infected people
*PTP*	Phylodynamic transmission parameter	-	Number of infections per year
*T*	Generation time	Average length of time between primary and secondary infections	Years
*γ*	Recovery rate from the disease	-	Number of persons per year
*μ*	Death rate of the population	-	Number of persons per year
*SSE*	Superspreading Events	Minimum expected number of secondary infections from a superspreader	Number of secondary infections
*k*	Dispersion parameter of the negative binomial distribution	-	-
*superspreader*	Top 1% of infected individuals when we rank them by their attributed secondary infections	-	-

In previous work, variation in the number of secondary infections per infected individual, *Z*, has been represented by a negative binomial distribution that is described by two parameters, (i) mean *R*_0_ among infections and (ii) the dispersion parameter *k* [8], [9]. A small *k* (<0.1) indicates that a small proportion of infected individuals actively transmit the pathogen, whilst a large *k* (>4) means that all infected individuals contribute approximately equally to onwards transmission [8], [10]. Lloyd-Smith et al. introduced a definition of superspreaders as the top 1% of hosts when ranked by the number of secondary infections they create [8]. Although superspreading events (SSE) (i.e. the minimum number of secondary infections generated by a superspreader) have been estimated for directly-transmitted acute infections [8], they have never been described for chronic viral infections. The indolent and subclinical nature of chronic infections makes it difficult to track primary and secondary infections of the multiple strains that concurrently transmit in a given population. The problem is further compounded for HIV and the hepatitis C virus (HCV) that circulate in socially-marginalised groups such as injecting drug users (IDUs) and commercial sex workers.

In addition to *R*_0_ and the variation in onward transmission, another epidemiologically-important parameter is the average time between the primary and secondary infections, typically termed the infection generation time (*T*; several other definitions are used in the literature). A short *T* indicates rapid transmission, whilst a longer *T* suggests slower spread but also longer carriage. The duration of carriage of pathogens, which is usually known, represents an upper-limit on *T* and thus it is reasonable to conclude that directly transmitted acute infections have *T*<1 month whilst chronic infections have *T* values on the order of months or years.

Here we show how transmission variability and infection generation time can be estimated by combining viral genomic data with surveillance data and mathematical epidemiology.

## Results/Discussion

### Conceptual modelling framework

The concept of effective population size (*Ne*) has been used in population genetics for at least 50 years (for a brief review see Text S1) [11], [12]. *Ne*(*t*) is generally defined as the size of an idealised population (one without selection or population structure) that experiences the same level of genetic drift as the studied population at time *t*. *Ne*(*t*) is typically lower than *N*(*t*), the population's actual size at time *t*. The ratio *N*(*t*)/*Ne*(*t*) thus indicates how similarly the real population's reproduction matches the assumptions of the idealised model [13], [14]. Under a wide range of scenarios this ratio represents the variation in offspring numbers among individuals [15], [16].

If the population in question is a viral epidemic, then *N*(*t*) is the number of infections at time *t* (or number of prevalent cases) and *Ne*(*t*) represents the effective number of infections (i.e. the number of infections of an idealised epidemic that experiences the same level of genetic drift as the studied population). Crucially, if genetic variation among strains has little or no effect on their ability to infect hosts, as appears to be the case for HIV and HCV [11] then the ratio *N*(*t*)/*Ne*(*t*), is formally equal to var(*Z*), the variance in the number of secondary infections [17], [18]:
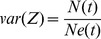
(1)
*N*(*t*) can be directly observed or estimated from surveillance data using classical epidemiological methods [19]. *Ne*(*t*) can be estimated by analysing the pattern of genetic diversity in a sample of the viral population. Specifically, methods based on coalescent theory, such as the skyline plot [11], [20], estimate the product of the coalescent *Ne*(*t*) multiplied by *T*, the generation time. The value var(Z)/T is inferable from empirical data and we here call it the phylodynamic transmission parameter, PTP. With all these estimates in hand it is therefore possible to estimate var(*Z*) from equation 1 as follows:
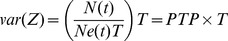
(2)PTP reflects two important features of the intensity of transmission within a population, (i) the variance of secondary infections among infections, and (ii) time between infections. Equation 2 suggests that an epidemic with a specific PTP is equally well described either by slow and highly variable onward transmission or by fast and more homogeneous onward transmission. This means that by comparing prevalent cases and genetic diversity (as measured by the skyline plot) alone, we cannot directly infer var(*Z*) and *T*; more information is required to separate these parameters. In the next two sections we consider practical aspects of inferring these two variables.

### Infection generation time

Volz and Frost [21], [22] incorporated mathematical epidemiology in coalescent models assuming that pathogens spread in the population according to compartmental models of epidemic spread. As theory predicts they showed that there is no constant transformation from *NeT* to *N* because as susceptible hosts decline in the population, *T* expands; a constant transformation from *NeT* to *N* is observed when the epidemic is on the exponential phase (i.e. *T* remains constant). Koelle and Rasmussen [23] showed similarly that a linear constant transformation of *NeT* to *N* is also observed when the epidemic is within a steady endemic state. Thus, if we compare *NeT* with *N* at the exponential phase or the endemic state we can assume that *T* remains constant.

### Distributions of numbers of secondary infections for epidemics with active and inactive transmitters

To describe the variability in onward transmission we require a probability density function of the random variable *Z*, the number of secondary infections per infected individual. Previous work has modeled variation in this number with a negative binomial distribution described by two parameters, mean *R*_0_ and a dispersion parameter *k* [8], [9]. Chronic viral infections, such as those caused by HIV and HCV, are unlikely to be well described by a single distribution. For these epidemics a significant proportion of transmissions result in inactive infections that transmit the virus no further and thus a mixed distribution is a more realistic representation.

In our study we define a sub-population of “inactive” infections whose expected number of secondary infections is equal to 0. The rest of the population is defined as “active”. Active infections comprise a proportion *u* of all infections and their expected number of secondary infections are assumed to be Poisson distributed with mean *R*_0,a_. The distribution of the number of secondary infections *Z* in the whole population (active and inactive combined) is therefore a zero-inflated Poisson distribution, such that:
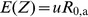
(3)

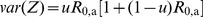
(4)
Equations 3 and 4 can be used to estimate the number of secondary infections of active infections (*R*_0,a_) provided that estimates of *E*(*Z*), *u* and var(*Z*) are available.

### Proof of concept: Concurrent nationwide epidemics of HCV

Well-described cohorts of HCV infections (of subtypes 1a, 1b, 3a and 4a) have been described in Greek populations [24], [25]. Crucially, for these epidemics we have both surveillance information and concurrent samples of viral genome sequences from the same population. First, we used inferred HCV incidence and prevalence by subtype from previous studies [25]. Next, we used the skyline plot method to estimate the value *Ne*(*t*)*T* for each subtype from the viral genome sequences sampled concurrently from the same populations (see Table S1) [26]–[28].

For both methods we assume that the population corresponds to the set of individuals chronically infected with HCV. The majority of patients with HCV infection develop persistent or chronic infection (60–92%) whilst a minority clears HCV-RNA (8–40%); viral clearance is much faster within the first 2 years of infection and slower thereafter (≪1% per year), while increased rates of viral clearance are associated with younger age, female gender, lack of HIV co-infection, chronic HBV infection and genetic variation in IL28B [29]–[42].

### HCV phylodynamic analysis

In total, 24, 27, 24 and 22 samples from Greek patients were amplified and sequenced for subtypes 1a, 1b, 3a and 4a, respectively (Table S1). The majority of subtype 1a and 3a infections were associated with injecting drug use, while for subtype 1b and 4a infections the source of infection was usually unknown. These distributions are consistent with previous epidemiological findings [24].

Phylogenetic trees (Figure S1) were estimated using a part of the NS5B region (nt 8297–8597) for which more reference sequences from other locations are available. These revealed the epidemics of different subtypes in Greece are not monophyletic and thus they arose through multiple introductions.

**Figure 1:**
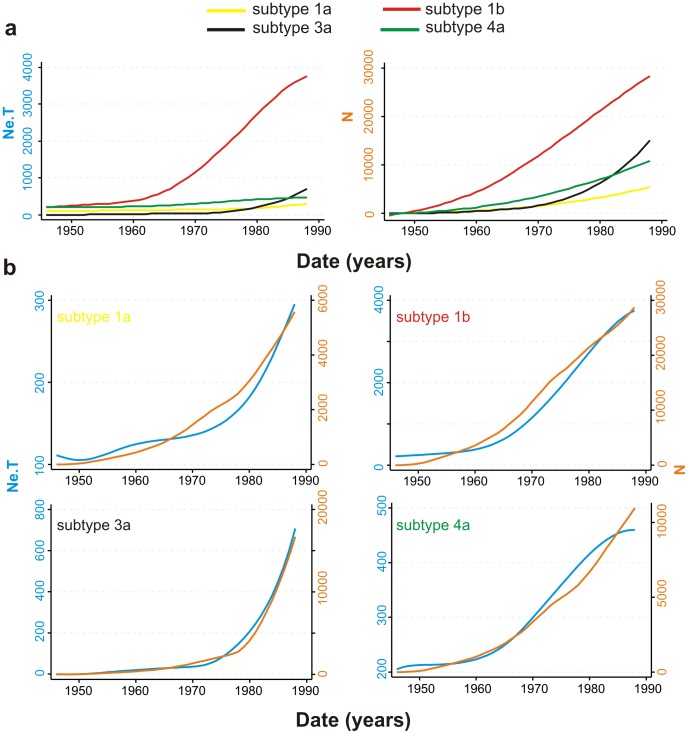
Plots through time of *NeT* (estimated from genetic data using the Bayesian skyline plot) versus *N* (estimated from surveillance data using back calculation). The plot of *N* is drawn by means of locally weighted smoothing on the scatter plot (lowess) of the estimated *N*. We have truncated the plots after 1990 as we wish to characterise HCV transmission prior the virus' discovery in 1989. The vertical axes of the plots through time of *NeT N* for each HCV subtype (B) have been scaled between maximum and minimum values.

Since the outbreaks were not monophyletic we can only provide upper limits of the date of introduction of each subtype (i.e. the date of the oldest possible introduction). Analysis using molecular clock coalescent methods (Figure 1, Figure S2) indicates that the 1a, 1b, 3a and 4a epidemics first entered the Greek population around 1965, 1958, 1975 and 1967, respectively (Table S2). It is important to note that the methods developed here depend on the exponential growth phase of each subtype, and not on the date of its most recent common ancestor, as the latter is more sensitive to sampling biases. The most striking difference in epidemic history among the subtypes is the rapid exponential growth of subtype 3a during 1978–1990, whereas the other subtypes appeared to expand more slowly during 1960–1990 (Figure 1).

### Epidemic and phylodynamic estimates are correlated

For each HCV subtype, the estimated plots of *N_e_*(*t*)*T* and *N*(*t*) for each subtype correspond with each other in relative size (Figure 1a), indicating that larger *N* corresponds to larger *N_e_T*. The plots of *N_e_*(*t*)*T* and *N*(*t*) for each subtype are also remarkably similar in shape (Figure 1b), indicating that PTP = (*N*(*t*)/*Ne*(*t*)*T*) is relatively constant through time. Subsequently, to estimate the ratio *N/N_e_T* for each subtype, we assessed the correlation of *N_e_T* and *N* during the period of exponential growth using linear regression (suppressing the constant term, since theory proposes that *N* is directly proportional to *N_e_*). The correlation of *N*(*t*) and *Ne*(*t*)*T* is thus given by *N*(*t*) = *a Ne*(*t*)*T*, such that *a* is an estimate of the phylodynamic transmission parameter PTP = (*N/NeT*). Since all these metrics are time-series data we corrected the cross-correlations between *NeT* and *N* for auto-correlation by means of the Newey-West method [43]. Specifically, we assessed the auto-correlation structure for each parameter and each subtype and then used the maximum lag between the cross-correlated data to correct statistical significance. Linear regressions of *N*(*t*) against *Ne*(*t*)*T* for each HCV subtype are strong and significant (p<0.01; R^2^ = 0.70–0.95). The regression gradients (*a*) provide estimates of *PTP* = (*N/NeT*), which vary from 15.6 to 43.4 for the different HCV subtypes (Table 2, S3).

**Table 2:** Estimates of transmission parameters for each HCV subtype.

	All				Transmitters	99^th^ percentile SSE
	PTP = (*N*/*NeT*)^1^ (95% C.I.)	E(*Z*) = *R_0_* (95% C.I.)	*T* 2	*u* 3	E(*Z*_a_) = Var(*Z*_a_) = *R_0_*_,a_	Top 1% (overall)^4^
**1a**	25.8 (21.2–30.2)	3.4 (3.3–3.5)	1.4	0.26	13.1	20
**1b**	15.6 (14.6–16.4)	4.5 (4.2–4.8)	20.6	0.06	75	83
**3a**	43.4 (38.6–48.2)	11.5 (10.7–12.4)	3.7	0.47	24.5	35
**4a**	27.8 (23.2–31.4)	2.4 (2.3–2.5)	0.9	0.2	12	18

1 The phylodynamic transmission parameter PTP = *N*/(*NeT*) has been estimated as the coefficient of the linear regression of *N* versus *NeT* without constant term. For the confidence intervals the autocorrelation structure of each variable has been taken into account according to the Newey-West correction.

2 Generation time estimated as Var(*Z*)/PTP (maximum estimate assuming that the minimum proportion of transmitters equals the proportion of IDUs in each subtype).

3 Proportion of transmitters, practically equal to the proportion of IDUs within each subtype.

4 Upper 1% of the distribution of secondary infections including transmitters and non-transmitters.

### Subtype-specific *R_0_* estimates

The subtype-specific estimates of mean *R*_0_ during the exponential growth phase of *Ne* or *N* were 2.4–11.5 (Table 2, Table S3) assuming that infectivity period is 40 years and life expectancy is 70 years. These estimates are similar to those reported previously for subtypes 1a and 1b (both global samples) and 4a (sampled from Egypt) [44]. The expansion of subtype 3a is characterised by faster epidemic growth over a shorter timeframe compared to the other subtypes (Figure 1) and this is reflected in the large *R*_0_ value for that subtype, which suggests an average of >10 secondary infections per primary infection.

### Model of secondary infections in the Greek HCV epidemics

Historically, HCV epidemics have taken two distinct forms: older transfusion and iatrogenic-related transmission, and more recent intravenous drug use-related (IDU-related) outbreaks. The earlier transmission was characterised by slower spread; individuals infected by transfusion or nosocomial transmission are less likely to practice high-risk behaviors and thus often represent transmission chain dead-ends. The more recent IDU-related epidemics are characterised by rapid spread. HCV is hyperendemic in IDUs worldwide with anti-HCV prevalence of 15–90% [45]; IDUs may share syringes, needles and other contaminated equipment and are likely to cause long transmission chains [46], [47]. As explained above, the *Z*-values of HCV epidemics are thus unlikely to be described well by a single distribution; instead we suggest a bimodal distribution model for the number of secondary infections (see Eq.3–5) that can represent both types of transmission behavior.

**Figure 2:**
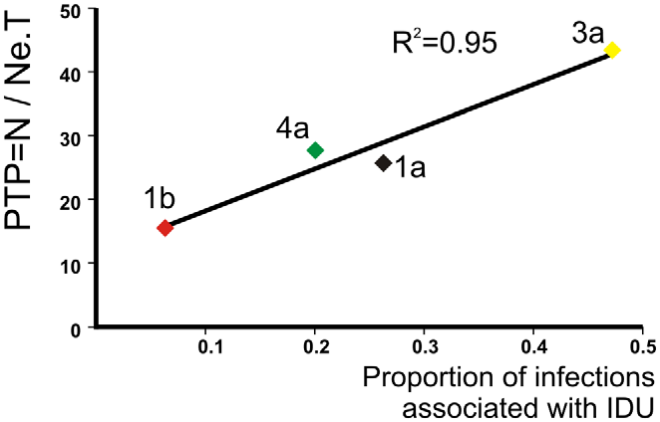
Scatter plot of the proportion of IDUs against the phylodynamic transmission potential ( = *N*/*NeT*) for each subtype.

We can use Equation 4 to test whether our model is congruent with epidemiological data. Equation 4 predicts that PTP increases with the proportion of “transmitters” in the population of infected individuals (provided that the proportion of transmitters is <50%, which is the case for all the HCV epidemics in this study). Regression of PTP against the percentage of IDU infections for each HCV subtype is strongly significant (Figure 2) whereas the regressions for other risk groups are not (Table S4). This suggests that the estimates of PTP are compatible with the known epidemiology of HCV. However, we note that this regression contains only 4 points and therefore data from more sub-epidemics are required to strengthen this finding.

### Estimation of the generation time (*T*)

There is no previously-available estimate for the generation time (*T*) of HCV since tracking of secondary infections is very difficult and date of infection is in most cases unknown. Some workers have suggested approximating *T* using the duration of infectiousness (1/(*γ*+*μ*)) [48], which for HCV is around 25 years (i.e 1/*γ* = 40 years and 1/*μ* = 70 years) (Table S3). If we assume that secondary infections follow a Poisson process within the duration of infectiousness (1/(*γ*+*μ*)) (i.e. if we perform a simulation of random secondary infections within 25 years of infectiousness), then the mean average time between primary and the subtending secondary infections is similarly high (∼12.5 years) regardless of the average number of secondary infections. Such values are epidemiologically and empirically unrealistic for many HCV epidemics: we know that IDUs usually get infected within 2 years after initiating injection [49].

**Figure 3:**
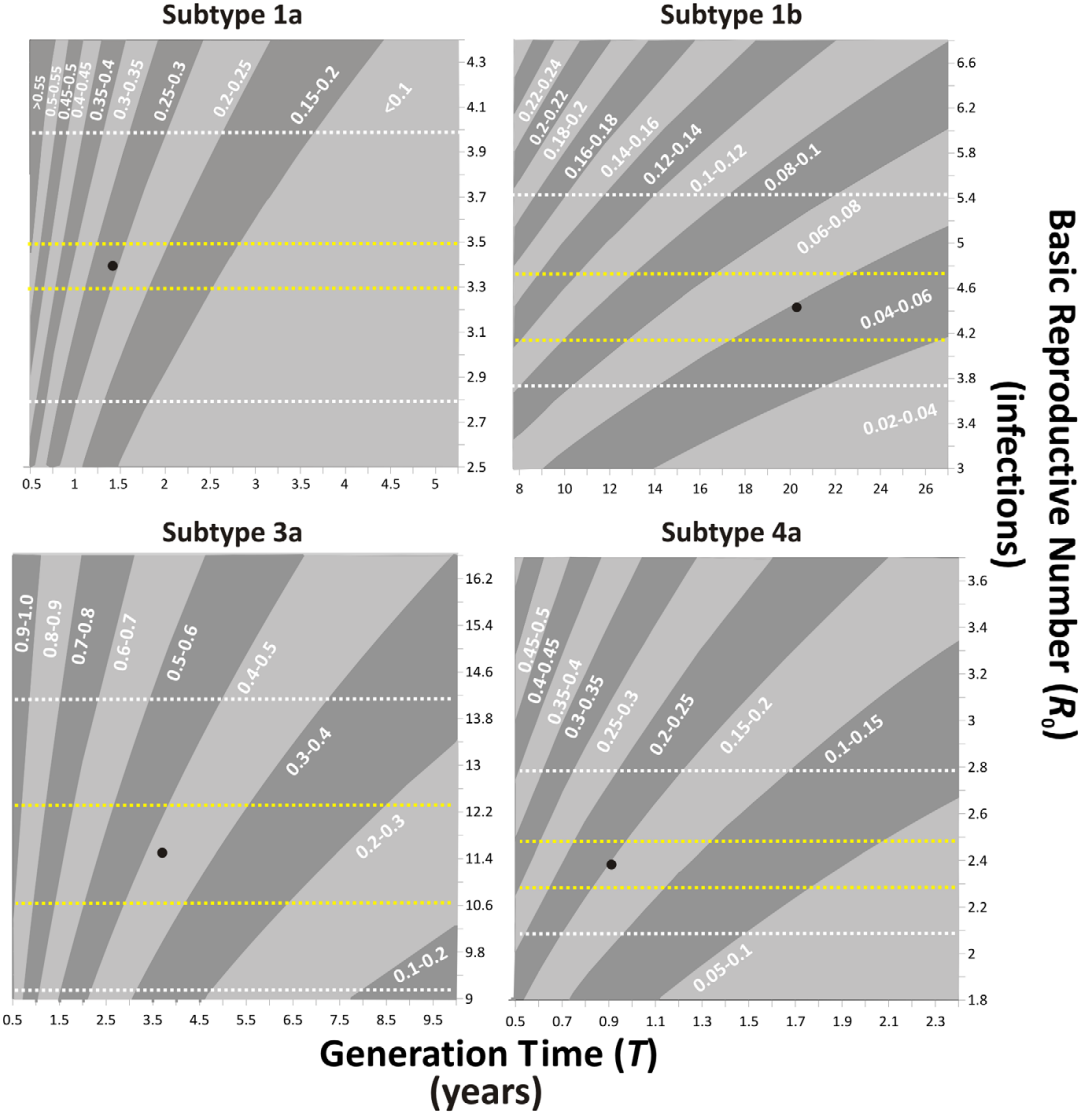
Contour plots showing how generation time (*T*), basic reproductive number (*R*_0_) and the proportion of transmitters in the population (*u*) co-vary. Gray bands highlight different values of *u*. The area between the white dashed lines represents *R*_0_ values estimated by sensitivity analysis of mortality and recovery rate (Table S3). The area between the yellow dashed lines represents the 95% confidence limits of *R*_0_ values estimated assuming 40 years of infectivity and 70 years of life expectancy. The black dots show the maximum *T* value for each subtype, which is defined by empirical values for *u* and the median values of *R*_0_ (see text).

By combining Equations 2, 3 4 taking into account that 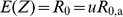 we can investigate how *T* is dependent on the proportion of the transmitters (*u*) and vice versa (Table 3, Figure 3):
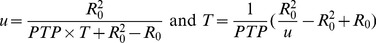
(5)


**Table 3:** Sensitivity analysis of the transmission parameters (var(*Z*), *u*, *R*_0,a_) accounting for different generation times (*T*) using the two-group (transmitter, non-transmitter) model of secondary infections (Eq.1).

	*R_0_*	*T*	var(*Z*)	*u*	*R*_0,a_
**1a**	3.4	1	25.8	0.34	9.99
		2	51.6	0.19	17.58
		10	258	0.04	78.28
		25	645	0.02	192.11
**1b**	4.5	1	15.6	0.65	6.97
		2	31.2	0.43	10.43
		10	156	0.12	38.17
		25	390	0.05	90.17
**3a**	11.5	1	43.4	0.81	14.27
		2	86.8	0.64	18.05
		10	434	0.24	48.24
		25	1085	0.11	104.85
**4a**	2.4	1	27.8	0.18	12.98
		2	55.6	0.1	24.57
		10	278	0.02	117.23
		25	695	0.01	290.98

The proportion of the transmitters (*u*) contrasted to the proportion of IDU, provides us information about epidemiologically probable generation times (*T*) i.e. we do not expect that the proportion of transmitters would be less than the proportion of IDU in the same population.

We assume that *T* is constant, which is reasonable for the exponential phase of the epidemic that we focus on [50]–[53]. Equation (5) shows that *T* is maximized at the smallest plausible value of *u*. The known epidemiology of HCV in IDUs suggests that the proportion of the transmitters (*u*) will not be smaller than the proportion of the IDUs (i.e. every IDU is likely to have transmitted), at least in our subtype 1a, 3a and 4a outbreaks, which are driven by intravenous drug use. Thus an epidemiologically-meaningful maximum *T* value can be obtained by setting *u* equal to the proportion of IDUs in the population (Figure 3).

Using Greek surveillance data on the proportion of HCV infections of each subtype associated with IDU [24] we estimate that the maximum *T* (Figure 3, Table 3) for subtype 1a (IDU: 26%) is 1.4 years, for subtype 3a (IDU: 47%) is 3.7 years and for subtype 4a (IDU: 20%) is 0.9 years. For the iatrogenic (non IDU-driven) epidemic of 1b (IDU:<10%) we estimate the maximum *T* close to the approximate duration of infectiousness (∼20 years) [Note that we use IDU as transmitters even if the epidemic is non-IDU driven; this is due to their engagement in repeated paid blood donation up to the end of the 1970s.] [54].

These estimates of *T* for subtypes 1a, 3a and 4a are more compatible with the natural history of the disease than those based on the duration of infectiousness (∼12.5 years). The probability of secondary infection per contact is expected to be higher during the first year of infection, when viral load is 10 times greater than later in infection [55], [56]. Also, in the first year patients are less likely to have ceased or reduced the high-risk behavior (e.g. IDU) that led them to be infected. Taken together, this suggests that secondary infections are more likely during the first year of infection. For subtype 1b the estimated *T* is artificially inflated due to its transmission route (see below).

### Analysing the transmission diversity of HCV epidemics

**Figure 4:**
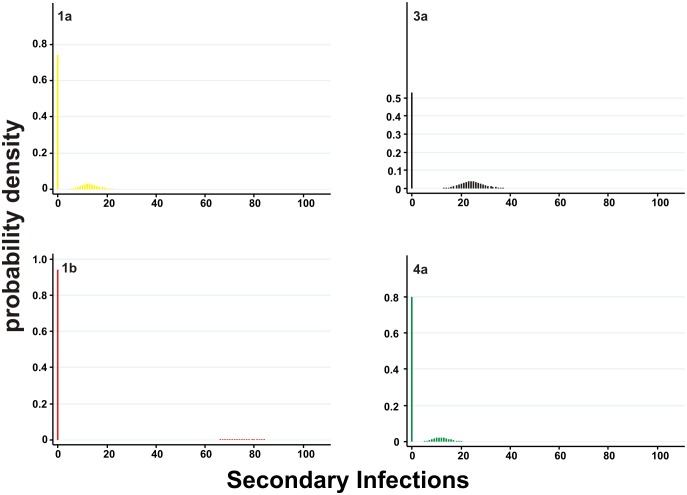
Estimated distributions of the number of secondary infections per primary infection for each HCV subtype.

**Figure 5:**
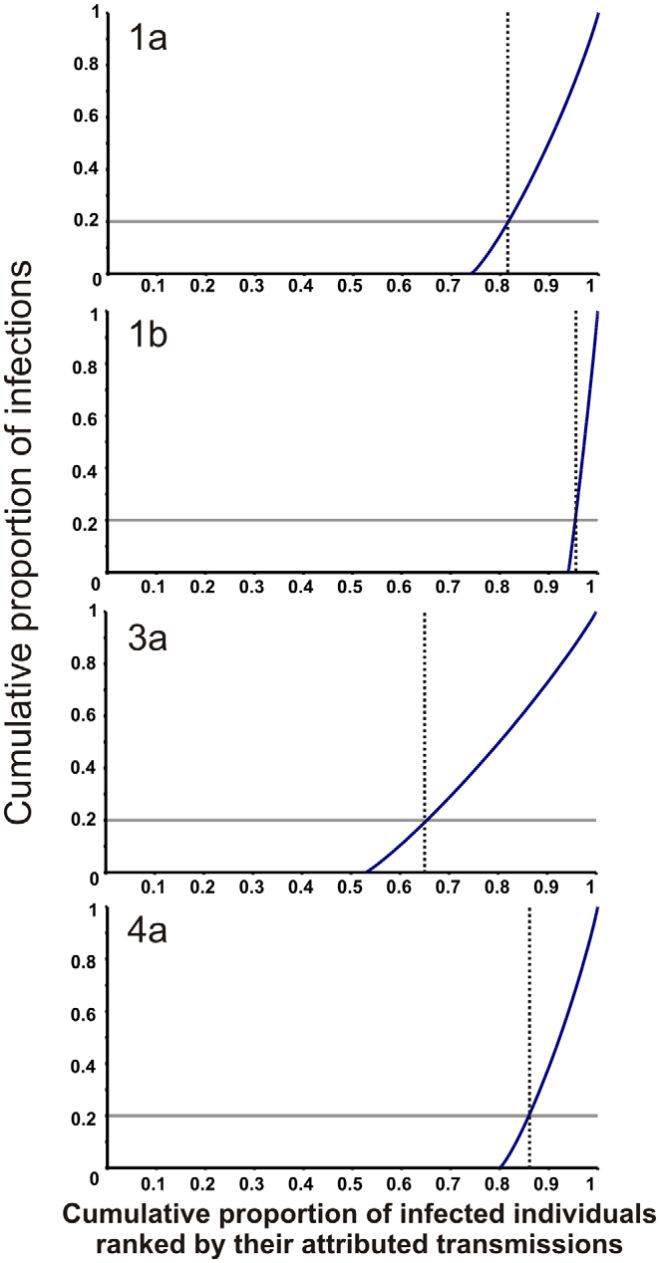
Cumulative proportion of onward infection versus the infected population ranked by the number of secondary infections they create. 20% of onward infections is indicated with a grey horizontal line. The proportion of the population that generates 80% of onward infections is shown by a vertical dashed line. HCV subtype 1a is close to the 80-20 rule (i.e. 80% of the infections are caused by the most infectious 18%).

We used equations (3) and (4) to estimate the basic reproductive number of the transmitters (*R*_0,a_) and the variability in onward transmission, given the values for *u*, PTP, *R*_0_ and *T* obtained above (Table 2). We estimate that for HCV subtypes 1a, 1b, 3a and 4a the *R*_0,a_ values ranged from 12 to 74 and the 99^th^ percentile SSE from 18 to 83 secondary infections (Table 2, Figure 4, Figure S4). Compared to directly-transmitted pathogens, HCV epidemics generally have large 99^th^ percentile SSE values, at least at the levels of SARS and Smallpox. For outbreaks of subtypes 1a, 1b, 3a and 4a investigated here, we estimate that 80% of the infections are caused by approximately 20%, 5%, 35% and 15% of the most infectious individuals, respectively (Figure 5).

The subtype 1b epidemic is the oldest and most prevalent in Greece, characterised by a small proportion of IDUs (6%) and was spread due to the use of contaminated blood and blood products. The very large number of secondary infections for each member of the transmitter population (*R*_0,a_ = 75), the high degree of superspreading (SSE 99^th^ percentile = 83) and the long generation time (*T*∼20 years) are compatible with the expected transmission dynamics of blood transfusions in the 1960s and 1970s. Historically, subtype 1b infections in Greece are attributed to the use of imported pooled plasma products, a practice that increased the probability of contaminating dozens of individuals from a single contaminated batch; the plasma products could be stored and distributed over many years leading to an artificially large “generation time”. Moreover, within Greece, infected IDUs during the 1960s and 1970s practiced repeated paid blood donations as a source of income. The reported dynamics of HCV-1b are typical of older (pre-1990s) HCV epidemics and do not apply to contemporary transmission (except in rare instances when transfusion safety breaks down. Similar trends in blood transfusion as a risk factor for HCV have been documented in many developed countries [46], [57]–[60].

On the other hand, the epidemics of subtypes 1a, 3a and 4a epidemics have higher proportions of IDUs (26%, 47% and 20% respectively) [24] and are typical of the modern HCV epidemics in the Western societies. For these epidemics the higher proportion of IDUs resulted in almost proportionally higher mean and variance in the number of secondary infections. The dynamics of these epidemics are still operating in the developed world and the estimated transmission parameters can be used to design mitigating strategies.

### Limitations of the study

Phylogenetic analysis suggests the sub-epidemics of HCV in Greece are the result of multiple introductions (i.e. non-monophyletic; Figure S1) suggesting that estimates of *Ne(t)T* near the root of the each subtype phylogeny may be biased upwards (because lineages fail to coalesce due to population structure). Two arguments suggest this is not a significant issue in our analysis. First, the trajectories of *N(t)* and *Ne(t)T*, which were estimated from separate data sources, closely correspond in four independent epidemics (in scale and shape) and *N* was obtained from epidemiological surveillance data of wholly Greek origin. Second, it is reasonable to assume that coalescent events within the exponential phase (the period during which we compared *N(t)* and *Ne(t)T*) did occur within Greece. That is, coalescences close to the root of each phylogeny (which may represent transmission outside Greece) were not used in our analysis. In the worst case scenario – that *Ne(t)T has* been overestimated – our estimate of PTP can be considered a lower bound and that variation in onward transmission might be even greater than reported here.

A second limitation of our study is that our estimate of PTP does not incorporate statistical uncertainty in the estimation of *N(t)* and *Ne(t)T*. In the future, we aim to develop a Bayesian approach to incorporate both sources of uncertainty and provide a proper posterior distribution for PTP.

Our approach provides information about superspreading from analytical relationships between the rate of coalescence (Ne), viral generation time (T), and prevalence (N) and thus is independent of phylogenetic topology. It is therefore complementary to alternative approaches that investigate how non-random contact structures affect the topology of a transmission tree [61]. At this point we should emphasize that further exploration and extension of the approach is required. For example a zero-inflated Poisson distribution of secondary infections does not fit most of the HIV-1 epidemics. A power-law distribution resulting from sexual-contact analysis would provide a more realistic approximation, for which a detailed analysis of the effect of network structure on PTP needs to be performed. Finally, simulation studies could explore the robustness of the approach under a wider range of epidemiologic scenarios, whilst larger datasets could empirically replicate our findings to support wider applicability of this approach e.g. to inform Public Health policies.

### Conclusion

We have shown that phylodynamic methods can be combined with epidemiological surveillance data to estimate the variability in ongoing transmission of a chronic viral epidemic, and to investigate its generation time. Both parameters are critical to the design of effective control measures but are very difficult to estimate from surveillance data alone. We tested the framework on a well-characterised set of HCV epidemic in Greece, showing that the results are epidemiologically coherent and suggesting that this approach could be a new tool for public health. We expect our approach to be most readily adapted to other chronic viral diseases such as HIV, but could also be applied to directly transmitted (e.g. Influenza) or vector-borne (e.g. Dengue) viral epidemics, for which superspreading events and generation times are largely unknown.

## Methods

### Ethics statement

Study approval was granted by the IRB of Athens University Medical School.

### Estimation of chronic HCV incidence and prevalence through time

The overall and genotype-specific incidence of chronic HCV infection has been estimated in previous studies using back-calculation [24], [25]. Briefly, the distribution of transmission risk groups among HCV infected individuals was obtained from 943 Greek patients enrolled in treatment studies [24], [25]. Enrolment took place between 1995 and 2000; patients were adults (18–70 years old) with a histological diagnosis of chronic hepatitis. Injecting drug use, transfusion, other and sporadic transmissions were reported by 24%, 32%, 6% and 38% of the patients, respectively. The distribution of the dates of infection within each transmission group was determined using data from 456 Greek patients enrolled in treatment studies with known dates of infection. We extended the back-calculation approach to estimate subtype-specific incidence of chronic HCV [25] in Greece as follows: a) we estimated the number of individuals infected with HCV in Greece, b) we obtained the distribution of HCV subtypes by year of onset for each transmission group within the infected population and c) we calculated subtype-specific incidence according to transmission group using the number of new infections in the past for each transmission group and the corresponding distribution of HCV subtypes by year of infection. The estimates for each transmission group were then combined to obtain an estimate of the overall genotype-specific incidence and prevalence during 1940–1990.

### HCV sequence data

Correct sampling is crucial to the inference of epidemic history from genetic data [62]. All available 1a, 1b, 3a and 4a subtype samples from distinct HCV-infected patients, tested within a 12-year period (1994–2006), were sorted according to their sampling dates, and at least one sample was randomly selected and sequenced for every 6-month interval. For cases in which no sample was available in a specific 6-month interval, the closest sample to that period was selected. Besides the sampling date, additional information was recorded for each sample: patient's age, sex, transmission group and treatment history (Table S1). Samples were excluded where the patient had a prior history of antiviral therapy and/or HIV co-infection, since these factors are believed to affect the intrahost evolution of the virus, thus (theoretically) introducing a bias into the estimation of substitution rate [63]. Sequencing of the HCV E2P7NS2 and NS5B regions was performed as previously described [26].

### Estimation of basic reproductive number (*R*_0_)

We estimated *R*_0_ assuming that the population is large enough to follow a deterministic Susceptible-Infected-Removed model (SIR) [3]:
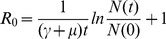
(6)where *N*(*t*) is the number of infected people at time *t* (prevalent cases), *N*(0) is the number of infected people at the baseline of the exponential growth phase, *γ* is the recovery rate of the disease and *μ* is the death rate in the general population. This equation is valid for the exponential phase of the epidemic growth. To estimate subtype-specific *R*_0_ we used the nl routine in STATA to fit the above equation to the estimated *N(t)* curve during the exponential growth phase, assuming an average life expectancy (1/*μ*) of 70 years and an average infectivity period (1/*γ*) of 40 years (i.e. excluding host mortality), which are plausible estimates for the study population (Table S3). Note that if the *N*(*t*) and *Ne*(*t*) are highly correlated (such that *N*(*t*)/*N*(0) is equal to *Ne*(*t*)/*Ne*(0)) then equation 6 shows that we can get equivalent estimates of *R*_0_ from the skyline plot..

### Identification of the exponential growth phase

To identify the exponential growth phase of each Greek HCV epidemic, we first defined the end of the exponential phase as 1990, to reflect the introduction of anti-HCV screening after the virus' discovery in 1989. The start of the exponential phase was detected using two methods. First, by visually inspecting the epidemic time series and selecting the first time point after 6 years of consecutive increases of *N* or *NeT*. Second, we employed a previously-published algorithm used in quantitative PCR experiments, where the identification of the exponential phase of a growth curve is crucial [64]. Both methods provided closely similar results (±3 years).

## Supporting Information

Figure S1 Phylogenetic trees (midpoint rooted) of the Greek isolates (blue circles) along with a global sample (all published sequences available at April 1^st^, 2010) on NS5B (nt 8297–8597).
(TIF)

Figure S2 Upper and lower limits of the 95% Higher Posterior Density (HPD) of the skyline plots (*NeT*) and of the 95% Confidence Intervals (C.I.) of the back-calculated number of prevalent cases (*N*).
(TIF)

Figure S3 Scatter plots of *N* against *NeT* for the exponential growth phase along with the fitted regression line that passes from the origin of the axis (i.e. suppressing the constant term). Note that regression has been performed correcting for auto-correlation according to the Newey-West method. We note an apparent deviation from linearity due to stochastic noise independently present the auto-correlated series. This deviation disappears when only independent data points are included in the plot.
(TIF)

Figure S4 Cumulative distribution of the secondary infections for the Greek HCV epidemics (solid lines) and directly transmitted pathogens (dashed lines) based on estimates provided by Lloyd-Smith et al. [30]. (SSE = Superspreading events)
(TIF)

Table S1 **A.** Demographic features and experimental efficiency in the sample used for the phylodynamic analysis, **B.** Demographic features of the patients used for the epidemiological analysis.
(PDF)

Table S2 Estimated parameters of the phylodynamic analysis.
(PDF)

Table S3 Sensitivity analysis for the estimated medians of the Basic Reproductive Numbers (*R*_0_).
(PDF)

Table S4 Regression analysis of the percentage of the risk group per genotype with the spread metrics PPT and *R*_0_ per genotype in the study population: coefficients of determination (Pearson's R^2^) are shown with associated level of significance (P value).
(PDF)

Text S1 Supplementary information.
(DOC)

## References

[1] Grassly NC, Fraser C (2008) Mathematical models of infectious disease transmission. Nat Rev Microbiol 6: 477–487. 18533288 10.1038/nrmicro1845 PMC7097581

[2] Anderson RM, May RM (1992) Infectious Diseases of Humans: Dynamics and Control. Oxford: Oxford University Press.

[3] Keeling MJ, Rohani P (2008) Modeling Infectious Diseases in Humans and Animals. Princeton, New Jersey: Princeton University Press.

[4] Kermack WO, McKendrick AG (1927) A contribution to the mathematical theory of epidemics. Proc R Soc Lond A 115: 700–721.

[5] Anderson RM, May RM (1979) Population biology of infectious diseases: Part I. Nature 280: 361–367. 460412 10.1038/280361a0

[6] May RM, Anderson RM (1987) Transmission dynamics of HIV infection. Nature 326: 137–142. 3821890 10.1038/326137a0

[7] Woolhouse ME, Dye C, Etard JF, Smith T, Charlwood JD, et al (1997) Heterogeneities in the transmission of infectious agents: implications for the design of control programs. Proc Natl Acad Sci U S A 94: 338–342. 8990210 10.1073/pnas.94.1.338 PMC19338

[8] Lloyd-Smith JO, Schreiber SJ, Kopp PE, Getz WM (2005) Superspreading and the effect of individual variation on disease emergence. Nature 438: 355–359. 16292310 10.1038/nature04153 PMC7094981

[9] Lloyd-Smith JO (2007) Maximum likelihood estimation of the negative binomial dispersion parameter for highly overdispersed data, with applications to infectious diseases. PloS one 2: e180. 17299582 10.1371/journal.pone.0000180 PMC1791715

[10] Garske T, Rhodes CJ (2008) The effect of superspreading on epidemic outbreak size distributions. J Theor Biol 253: 228–237. 18423673 10.1016/j.jtbi.2008.02.038

[11] Grenfell BT, Pybus OG, Gog JR, Wood JL, Daly JM, et al (2004) Unifying the epidemiological and evolutionary dynamics of pathogens. Science 303: 327–332. 14726583 10.1126/science.1090727

[12] Wright S (1938) Size of population and breeding structure in relation to evolution. Science 87: 430–431.

[13] Hedrick P (2005) Large variance in reproductive success and the Ne/N ratio. Evolution 59: 1596–1599. 16153045

[14] O'Dea EB, Wilke CO (2011) Contact heterogeneity and phylodynamics: how contact networks shape parasite evolutionary trees. Interdisciplinary perspectives on infectious diseases 2011: 238743. 21151699 10.1155/2011/238743 PMC2995904

[15] Kimura M, Crow JF (1963) The measurement of effective population number. Evolution 17: 279–288.

[16] Felsenstein J (1971) Inbreeding and variance effective number in populations with overlapping generations. Genetics 68: 581–597. 5166069 10.1093/genetics/68.4.581 PMC1212678

[17] Tavare S, Balding DJ, Griffiths RC, Donnelly P (1997) Inferring coalescence times from DNA sequence data. Genetics 145: 505–518. 9071603 10.1093/genetics/145.2.505 PMC1207814

[18] Kingman JFC (1982) On the genealogy of large populations. J App Prob 19A: 27–43.

[19] Deuffic S, Buffat L, Poynard T, Valleron AJ (1999) Modeling the hepatitis C virus epidemic in France. Hepatology 29: 1596–1601. 10216148 10.1002/hep.510290528

[20] Strimmer K, Pybus OG (2001) Exploring the demographic history of DNA sequences using the generalized skyline plot. Mol Biol Evol 18: 2298–2305. 11719579 10.1093/oxfordjournals.molbev.a003776

[21] Frost SD, Volz EM (2010) Viral phylodynamics and the search for an ‘effective number of infections’. Philos Trans R Soc Lond B Biol Sci 365: 1879–1890. 20478883 10.1098/rstb.2010.0060 PMC2880113

[22] Volz EM, Kosakovsky Pond SL, Ward MJ, Leigh Brown AJ, Frost SD (2009) Phylodynamics of infectious disease epidemics. Genetics 183: 1421–1430. 19797047 10.1534/genetics.109.106021 PMC2787429

[23] Koelle K, Rasmussen DA (2012) Rates of coalescence for common epidemiological models at equilibrium. J R Soc Interface 9: 997–1007. 21920961 10.1098/rsif.2011.0495 PMC3306638

[24] Katsoulidou A, Sypsa V, Tassopoulos NC, Boletis J, Karafoulidou A, et al (2006) Molecular epidemiology of hepatitis C virus (HCV) in Greece: temporal trends in HCV genotype-specific incidence and molecular characterization of genotype 4 isolates. J Viral Hepat 13: 19–27. 16364078 10.1111/j.1365-2893.2005.00649.x

[25] Sypsa V, Touloumi G, Tassopoulos NC, Ketikoglou I, Vafiadis I, et al (2004) Reconstructing and predicting the hepatitis C virus epidemic in Greece: increasing trends of cirrhosis and hepatocellular carcinoma despite the decline in incidence of HCV infection. J Viral Hepat 11: 366–374. 15230860 10.1111/j.1365-2893.2004.00517.x

[26] Magiorkinis G, Magiorkinis E, Paraskevis D, Ho SY, Shapiro B, et al (2009) The global spread of hepatitis C virus 1a and 1b: a phylodynamic and phylogeographic analysis. PLoS Med 6: e1000198. 20041120 10.1371/journal.pmed.1000198 PMC2795363

[27] Drummond AJ, Ho SY, Phillips MJ, Rambaut A (2006) Relaxed phylogenetics and dating with confidence. PLoS Biol 4: e88. 16683862 10.1371/journal.pbio.0040088 PMC1395354

[28] Drummond AJ, Rambaut A (2007) BEAST: Bayesian evolutionary analysis by sampling trees. BMC Evol Biol 7: 214. 17996036 10.1186/1471-2148-7-214 PMC2247476

[29] Zhang M, Rosenberg PS, Brown DL, Preiss L, Konkle BA, et al (2006) Correlates of spontaneous clearance of hepatitis C virus among people with hemophilia. Blood 107: 892–897. 16204310 10.1182/blood-2005-07-2781 PMC1895891

[30] Vogt M, Lang T, Frosner G, Klingler C, Sendl AF, et al (1999) Prevalence and clinical outcome of hepatitis C infection in children who underwent cardiac surgery before the implementation of blood-donor screening. N Engl J Med 341: 866–870. 10498458 10.1056/NEJM199909163411202

[31] Tillmann HL, Thompson AJ, Patel K, Wiese M, Tenckhoff H, et al (2010) A polymorphism near IL28B is associated with spontaneous clearance of acute hepatitis C virus and jaundice. Gastroenterology 139: 1586–1592. 20637200 10.1053/j.gastro.2010.07.005

[32] Thomas DL, Thio CL, Martin MP, Qi Y, Ge D, et al (2009) Genetic variation in IL28B and spontaneous clearance of hepatitis C virus. Nature 461: 798–801. 19759533 10.1038/nature08463 PMC3172006

[33] Thomas DL, Astemborski J, Rai RM, Anania FA, Schaeffer M, et al (2000) The natural history of hepatitis C virus infection: host, viral, and environmental factors. JAMA 284: 450–456. 10904508 10.1001/jama.284.4.450

[34] Seeff LB, Miller RN, Rabkin CS, Buskell-Bales Z, Straley-Eason KD, et al (2000) 45-year follow-up of hepatitis C virus infection in healthy young adults. Ann Intern Med 132: 105–111. 10644270 10.7326/0003-4819-132-2-200001180-00003

[35] Santantonio T, Medda E, Ferrari C, Fabris P, Cariti G, et al (2006) Risk factors and outcome among a large patient cohort with community-acquired acute hepatitis C in Italy. Clin Infect Dis 43: 1154–1159. 17029134 10.1086/507640

[36] Kenny-Walsh E (1999) Clinical outcomes after hepatitis C infection from contaminated anti-D immune globulin. Irish Hepatology Research Group. N Engl J Med 340: 1228–1233. 10210705 10.1056/NEJM199904223401602

[37] Farci P, Alter HJ, Wong D, Miller RH, Shih JW, et al (1991) A long-term study of hepatitis C virus replication in non-A, non-B hepatitis. N Engl J Med 325: 98–104. 1646962 10.1056/NEJM199107113250205

[38] Conry-Cantilena C, VanRaden M, Gibble J, Melpolder J, Shakil AO, et al (1996) Routes of infection, viremia, and liver disease in blood donors found to have hepatitis C virus infection. N Engl J Med 334: 1691–1696. 8637513 10.1056/NEJM199606273342602

[39] Bortolotti F, Verucchi G, Camma C, Cabibbo G, Zancan L, et al (2008) Long-term course of chronic hepatitis C in children: from viral clearance to end-stage liver disease. Gastroenterology 134: 1900–1907. 18439604 10.1053/j.gastro.2008.02.082

[40] Alter MJ, Margolis HS, Krawczynski K, Judson FN, Mares A, et al (1992) The natural history of community-acquired hepatitis C in the United States. The Sentinel Counties Chronic non-A, non-B Hepatitis Study Team. N Engl J Med 327: 1899–1905. 1280771 10.1056/NEJM199212313272702

[41] Alter MJ, Kruszon-Moran D, Nainan OV, McQuillan GM, Gao F, et al (1999) The prevalence of hepatitis C virus infection in the United States, 1988 through 1994. N Engl J Med 341: 556–562. 10451460 10.1056/NEJM199908193410802

[42] Alter HJ, Seeff LB (2000) Recovery, persistence, and sequelae in hepatitis C virus infection: a perspective on long-term outcome. Semin Liver Dis 20: 17–35. 10895429 10.1055/s-2000-9505

[43] Newey WK, West KD (1987) A Simple, Positive Semi-definite, Heteroskedasticity and Autocorrelation Consistent Covariance Matrix. Econometrica 3: 703–708.

[44] Pybus OG, Charleston MA, Gupta S, Rambaut A, Holmes EC, et al (2001) The epidemic behavior of the hepatitis C virus. Science 292: 2323–2325. 11423661 10.1126/science.1058321

[45] Nelson PK, Mathers BM, Cowie B, Hagan H, Des Jarlais D, et al (2011) Global epidemiology of hepatitis B and hepatitis C in people who inject drugs: results of systematic reviews. Lancet 378: 571–583. 21802134 10.1016/S0140-6736(11)61097-0 PMC3285467

[46] Alter MJ (2011) HCV routes of transmission: what goes around comes around. Semin Liver Dis 31: 340–346. 22189974 10.1055/s-0031-1297923

[47] Alter MJ, Hadler SC, Judson FN, Mares A, Alexander WJ, et al (1990) Risk factors for acute non-A, non-B hepatitis in the United States and association with hepatitis C virus infection. JAMA 264: 2231–2235. 2170702

[48] Wallinga J, Lipsitch M (2007) How generation intervals shape the relationship between growth rates and reproductive numbers. Proc Biol Sci 274: 599–604. 17476782 10.1098/rspb.2006.3754 PMC1766383

[49] Hagan H, Pouget ER, Des Jarlais DC, Lelutiu-Weinberger C (2008) Meta-regression of hepatitis C virus infection in relation to time since onset of illicit drug injection: the influence of time and place. Am J Epidemiol 168: 1099–1109. 18849303 10.1093/aje/kwn237 PMC2727245

[50] Raptopoulou-Gigi M, Orphanou E, Lalla TH, Lita A, Garifallos A (2001) Prevalence of hepatitis C virus infection in a cohort of pregnant women in northern Greece and transmission of HCV from mother to child. Eur J Epidemiol 17: 263–266. 11680545 10.1023/a:1017951605272

[51] Sypsa V, Hadjipaschali E, Hatzakis A (2001) Prevalence, risk factors and evaluation of a screening strategy for chronic hepatitis C and B virus infections in healthy company employees. Eur J Epidemiol 17: 721–728. 12086089 10.1023/a:1015671627577

[52] Goritsas C, Plerou I, Agaliotis S, Spinthaki R, Mimidis K, et al (2000) HCV infection in the general population of a Greek island: prevalence and risk factors. Hepato-Gastroenterology 47: 782–785. 10919032

[53] Cornberg M, Razavi HA, Alberti A, Bernasconi E, Buti M, et al (2011) A systematic review of hepatitis C virus epidemiology in Europe, Canada and Israel. Liver Int 31 Suppl 2: 30–60. 21651702 10.1111/j.1478-3231.2011.02539.x

[54] Nelson KE, Vlahov D, Margolick J, Bernal M, Taylor E (1990) Blood and plasma donations among a cohort of intravenous drug users. JAMA 263: 2194–2197. 1969502

[55] Page K, Hahn JA, Evans J, Shiboski S, Lum P, et al (2009) Acute hepatitis C virus infection in young adult injection drug users: a prospective study of incident infection, resolution, and reinfection. J Infect Dis 200: 1216–1226. 19764883 10.1086/605947 PMC2821203

[56] Cox AL, Netski DM, Mosbruger T, Sherman SG, Strathdee S, et al (2005) Prospective evaluation of community-acquired acute-phase hepatitis C virus infection. Clin Infect Dis 40: 951–958. 15824985 10.1086/428578

[57] Armstrong GL, Alter MJ, McQuillan GM, Margolis HS (2000) The past incidence of hepatitis C virus infection: implications for the future burden of chronic liver disease in the United States. Hepatology 31: 777–782. 10706572 10.1002/hep.510310332

[58] Williams IT, Bell BP, Kuhnert W, Alter MJ (2011) Incidence and transmission patterns of acute hepatitis C in the United States, 1982–2006. Arch Intern Med 171: 242–248. 21325115 10.1001/archinternmed.2010.511

[59] Alter HJ, Klein HG (2008) The hazards of blood transfusion in historical perspective. Blood 112: 2617–2626. 18809775 10.1182/blood-2008-07-077370 PMC2962447

[60] Chung H, Ueda T, Kudo M (2010) Changing trends in hepatitis C infection over the past 50 years in Japan. Intervirology 53: 39–43. 20068339 10.1159/000252782

[61] Leventhal GE, Kouyos R, Stadler T, Wyl V, Yerly S, et al (2012) Inferring epidemic contact structure from phylogenetic trees. PLoS Comput Biol 8: e1002413. 22412361 10.1371/journal.pcbi.1002413 PMC3297558

[62] Stack JC, Welch JD, Ferrari MJ, Shapiro BU, Grenfell BT (2010) Protocols for sampling viral sequences to study epidemic dynamics. J R Soc Interface 7: 1119–1127. 20147314 10.1098/rsif.2009.0530 PMC2880085

[63] Danta M, Semmo N, Fabris P, Brown D, Pybus OG, et al (2008) Impact of HIV on Host-Virus Interactions during Early Hepatitis C Virus Infection. J Infect Dis 11: 1558–1566. 10.1086/587843 18419344

[64] Tichopad A, Dilger M, Schwarz G, Pfaffl MW (2003) Standardized determination of real-time PCR efficiency from a single reaction set-up. Nucleic Acids Res 31: e122. 14530455 10.1093/nar/gng122 PMC219490

