# Supplementary material for: Integrating Phylodynamics and Epidemiology to Estimate Transmission Diversity in Viral Epidemics
Source: PLoS Comput Biol. 2013 Jan 31;9(1):e1002876. doi: 10.1371/journal.pcbi.1002876 (PMC3561042; doi:10.1371/journal.pcbi.1002876)
Supplement: Table S1 — A. Demographic features and experimental efficiency in the sample used for the phylodynamic analysis, B. Demographic features of the patients used for the epidemiological analysis. (PDF) [file pcbi.1002876.s005.pdf]

Table S1

A.

	**Subtype 1a**	**Subtype 1b**	**Subtype 3a**	**Subtype 4a**
**Samping period**	N=24	N=27	N=24	N=22
Range	1994-2003	1994-2004	1995-2006	1995-2004
Median	1998	2001	2001	1999
**Age (at sampling date)**				
Range	17-53	19-76	23-46	26-69
Median	33	54	38	40
**Sex, n(%)**				
Male	19(79)	14(52)	16(67)	13(59)
Female	5(21)	13(48)	8(33)	9(41)
**Risk group, n(%)**				
Transfusion	3(12.5)	6(22)	1(4)	3(14)
Hemophiliac	2(8)	0(0)	0(0)	0(0)
IDU	12(50)	2(7)	17(71)	2(9)
Other*	4(17)	3(11)	0(0)	1(5)
Unknown	3(12.5)	16(59)	6(25)	16(73)
**Succesful PCR/Sequencing**				
E2-P7-NS2	14	19	19	8
NS5B	23	22	21	18

*Sexual partner of HCV carrier, hospitalization

B.

	**Subtype 1a**	**Subtype 1b**	**Subtype 3a**	**Subtype 4a**
	N=206	N=537	N=445	N=209
**Age**				
Mean (SD)	39.4 (14.7)	50.0 (14.2)	37.5 (11.9)	43.3 (13.5)
**Sex, n(%)**				
Male	166 (16.0)	303 (29.2)	327 (31.5)	137 (13.2)
Female	40 (7.3)	234 (42.7)	118 (21.5)	72 (13.1)
**Risk group, n(%)**				
Transfusion	26 (12.6)	136 (25.3)	49 (11.0)	41 (19.6)
Hemophiliac	59 (28.6)	49 (9.1)	26 (5.8)	8 (3.8)
IDU	54 (26.2)	34 (6.3)	210 (47.2)	42 (20.1)
Other*	41 (19.9)	115 (21.4)	78 (17.5)	50 (23.9)
Unknown	26 (12.6)	203 (37.8)	82 (18.4)	68 (32.5)

*Sexual partner of HCV carrier, hospitalization, hemodialysis/renal transplantation, occupational exposure, tattoo
