# Supplementary material for: Integrating Phylodynamics and Epidemiology to Estimate Transmission Diversity in Viral Epidemics
Source: PLoS Comput Biol. 2013 Jan 31;9(1):e1002876. doi: 10.1371/journal.pcbi.1002876 (PMC3561042; doi:10.1371/journal.pcbi.1002876)
Supplement: Table S2 — Estimated parameters of the phylodynamic analysis. (PDF) [file pcbi.1002876.s006.pdf]

TableS2

Molecular clock	E2P7NS2 Relaxed log-normal	E2P7NS2 Strict	NS5B Relaxed log-normal	Combined Relaxed log normal	Median Dates of the combined estimation
**Subtype 1a**					
tMRCA (years)	35 (13-147)	35 (15-144)	39 (14-221)	38 (19-71)	1965
Rate ($x10^{-3}$) substitutions/site/year	2.7 (0.011 - 5.6)	2.7 (0.029 - 5.4)	1.4 (0.58 - 3.4)	1.9 (0.32 - 3.8)	
Coefficient of Variation	0.092		0.31	0.33	
**Subtype 1b**					
tMRCA	48 (24 - 125)	48 (27 - 99)	34 (17 - 115)	46 (22 - 64)	1958
Rate ($x10^{-3}$) substitutions/site/year	2.6 (0.56 - 4.6)	2.7 (0.92 -4.6)	1.99 (0.18 - 3.7)	2.24 (0.83 - 3.8)	
Coefficient of Variation	0.108		0.26	0.26	
**Subtype 3a**					
tMRCA	30 (19 - 50)	30 (22 - 42)	52 (16 - 188)	28 (19 - 40)	1975
Rate ($x10^{-3}$) substitutions/site/year	2.42 (1.13 - 3.73)	2.5 (1.46 - 3.5)	0.99 (0.11 - 2.24)	2.26 (1.33 - 3.45)	
Coefficient of Variation	0.116		0.44	0.58	
**Subtype 4a**					
tMRCA	69 (10-170)	32 (13-94)	62 (12-770)	36 (12-58)	1967
Rate ($x10^{-3}$) substitutions/site/year	2.5 (0.013 - 5.0)	2.5 (0.27 - 4.7)	0.73 (0.004 - 2.4)	1.8 (0.47 - 4.4)	
Coefficient of Variation	0.117		0.45	0.56	
