# Supplementary material for: Integrating Phylodynamics and Epidemiology to Estimate Transmission Diversity in Viral Epidemics
Source: PLoS Comput Biol. 2013 Jan 31;9(1):e1002876. doi: 10.1371/journal.pcbi.1002876 (PMC3561042; doi:10.1371/journal.pcbi.1002876)
Supplement: Table S3 — Sensitivity analysis for the estimated medians of the Basic Reproductive Numbers (R 0). (PDF) [file pcbi.1002876.s007.pdf]

Table S3

Subtype 1a

		Infectivity period		
	**Years**	**30**	**40**	**50**
**Life expectancy**	**60**	2.8	3.2	3.5
	**70**	2.9	3.4	3.7
	**80**	3.0	3.5	3.8
	**90**	3.1	3.6	4.0

Subtype 1b

		Infectivity period		
	**Years**	**30**	**40**	**50**
**Life expectancy**	**60**	3.8	4.3	4.8
	**70**	3.9	4.5	5.0
	**80**	4.0	4.7	5.3
	**90**	4.1	4.8	5.5

Subtype 3a

		Infectivity period		
	**Years**	**30**	**40**	**50**
**Life expectancy**	**60**	9.2	10.9	12.2
	**70**	9.6	11.5	13.0
	**80**	10.0	12.0	13.7
	**90**	10.3	12.4	14.2

Subtype 4a

		Infectivity period		
	**Years**	**30**	**40**	**50**
**Life expectancy**	**60**	2.1	2.3	2.5
	**70**	2.1	2.4	2.6
	**80**	2.2	2.5	2.7
	**90**	2.2	2.5	2.8
