# Supplementary material for: Integrating Phylodynamics and Epidemiology to Estimate Transmission Diversity in Viral Epidemics
Source: PLoS Comput Biol. 2013 Jan 31;9(1):e1002876. doi: 10.1371/journal.pcbi.1002876 (PMC3561042; doi:10.1371/journal.pcbi.1002876)
Supplement: Table S4 — Regression analysis of the percentage of the risk group per genotype with the spread metrics PPT and R 0 per genotype in the study population: coefficients of determination (Pearson's R2) are shown with associated level of significance (P value). (PDF) [file pcbi.1002876.s008.pdf]

Table S4

		PPT=N/(NeT)		R_0	
		R^2	P value	R^2	P value
Risk group (%)	**Iatrogenic**	0.43	0.34	0.83	0.1
	**Transfusion**	0.53	0.27	0.39	0.37
	**IDU**	0.95	0.02	0.60	0.22
	**Other**	0.31	0.45	0.08	0.71
