# Supplementary material for: Integrating Phylodynamics and Epidemiology to Estimate Transmission Diversity in Viral Epidemics
Source: PLoS Comput Biol. 2013 Jan 31;9(1):e1002876. doi: 10.1371/journal.pcbi.1002876 (PMC3561042; doi:10.1371/journal.pcbi.1002876)
Supplement: Text S1 — Supplementary information. (DOC) [file pcbi.1002876.s009.doc]

**Text S1:** Supplementary Information

**Table of Contents** *Page*

1. A brief review on the relationship between the effective population size (*Ne*) and the census population size (*N*) in population genetics 3

a) Effective population number (*Ne*) 3

b) The ratio of effective population size (*Ne*) against census population

size (*N*) (*Ne*/*N*) 5

c) Derivation of *Ne*/*N* for infected populations 7

2. Phylodynamics estimates of HCV epidemics in Greece 8

a) PCR – Sequencing 8

b) Phylogenetics – Phylodynamics 9

3. Supplementary References 11

**1. A brief review of the relationship between the effective population size (*Ne*) and the census population size (*N*) in population genetics**

*(a) Effective population number (Ne)*

In the history of population genetics there have been numerous approaches to defining effective population size or number. Most of them create a measurable quantitative metric for the size of an idealized population structure that has the same properties as the real population. The first realization of such a metric was by Sewall Wright, where effective population size is determined as the size of idealized population in which each individual has an equal expectation of progeny. This idealized population is known as the Wright-Fisher model, for which the number of progeny per parent is distributed according to a Poisson distribution.

Kimura provided two distinct definitions of the effective population number. The first, called the inbreeding effective number (*Ne_i_*), is defined as the population size of an idealized population having the same amount of inbreeding as the studied population. He derived a formula between the effective inbreeding number and the census population size *N* of a monoecious diploid population:

$Ne_{i}=\frac{4N-2}{V_{k}+2}$ (S.Eq.1)

Where *V_k_* is the variance in the number of progeny per parent.

In the same paper he describes a second definition of the effective population number, the variance effective number *Ne_v_*, which is the size of an idealized population having the same amount of random genetic drift as the studied population. He proves that when the population size is constant and there is random mating *Ne_v_* and *Ne_i_* have the same size. Furthermore for overlapping diploid generations Kimura provided a simple formula for the effective inbreeding size

$Ne_{i}=\frac{N}{b\tau}$(S.Eq.2)

Where *b* is the average birth rate and *τ* is the average age of reproduction.

Felsenstein provided a correction of the *Ne_i_* in Kimura’s initial proposal by deriving the general formula:

$Ne_{i}=\frac{N_{1}T}{1+\sum_{i=1}^{\infty} l_{i}s_{i}d_{i}v_{i+1}^{2}}$(S.Eq.3)

Where *N_1_* is the birth rate, *T* is the generation time, *N_1_T* is defined as the total reproductive value and the second term in the denominator is roughly the probability of death of an individual while it still has reproductive value. By applying this formula to the Moran model he proves that:

$Ne_{i}=\frac{N}{2-\frac{1}{N}}\approx_{N>>0} \frac{N}{2}$(S.Eq.4)

He also provides a proof that under a constant population model and random mating *Ne_v_* and *Ne_i_* have the same size.

A third realization of the effective population size, the coalescent effective size *Ne_c_*, is generally described as the size of the population estimated under the n-coalescence of Kingman . Wakeley and Sargsyan clarified that *Ne_c_* is an effective size which refers to the mutation rate as a property of the idealized population and provided a general formula for it:

$Ne_{c}=\frac{b_{n}}{c_{n}}$(S.Eq.5)

where *c_n_* is the probability that a pair of ancestral lineages are descended from a common ancestor and *b_n_* is the probability that a single ancestral lineage is newly born. They also demonstrate that for the Wright-Fisher model *Ne_c_*=*N* and that for the Moran model $Ne_{c}=\frac{N}{2}$. Thus, under the most widely used population models the three definitions of the effective population numbers (the inbreeding, the variance and the coalescence) converge.

*(b) The ratio of effective population size (N_e_) against census population size (N) (N_e_/N)*

As most of the definitions of effective population size refer to the size of an idealized population that has the same property as the studied population, the ratio of *N*_e_/*N* is a metric showing the departure of the real population from the idealized one. S.Eq.1 suggests that departure from the idealized population can be attributed to the significantly different variance in the number of progeny per parent from the one suggested by the Poisson distribution. The ratio *N_e_*/*N* has been used in this context to describe departures of the studied population from the idealized model, such as uneven sex ratio, variance in family sizes greater than the mean, and variance in reproductive success .

Hedgecock showed that in mollusks or fishes most of the generations are descended from a very low proportion of individuals. The phenomenon was attributed to a mixture of different environmental and reproductive activity factors. As a result *Ne*/*N* was very low and the phenomenon, compared to a sweepstake-like chance (due to the randomness of the very small proportion of individuals producing a very large number of offspring), was named “the Hedgecock effect”. Hedrick estimated that in an extreme case of such a phenomenon, that is only one individual contributes to the next generation, *Ne*/*N* should equal to 1/*N* and that generally in the sweepstake-like reproduction *Ne*/*N* should be approximately *Nb*/*N,* where *Nb* is the number of the breeding adults.

Wakeley and Sargsyan developed a coalescent model of which the Wright-Fisher, Moran and sweepstakes-like reproduction are special cases. They showed that generally the coalescent effective population size is given by the formula:

$Ne=\frac{Y_{N}}{\varphi}(1+o(1))$ (S.Eq.6)

where *Y_N_* is the number of potential parents of the offspring replacing the *X_N_* individuals that died in a disturbance event, *ϕ* is the fraction of the population removed in each disturbance event and *o*(1) goes to zero as *N→∞*. From that formula it can be easily shown that as *N→∞*

$\frac{Ne}{N}\mathop{=}\limits_{N\to\infty} \frac{Y_{N}}{X_{N}}$(S.Eq.7)

This formula shows that the ratio *Ne*/*N* is an estimate of the ratio of the individuals passing their genes divided by the number of the individuals dying. When *Y_N_*<<*X_N_* S.Eq.7 provides the same results as proposed by Hedrick and therefore it shows the proportion of individuals contributing to the next generation.

*(c) Derivation of Ne/N for infected populations*

The skyline plot (*18*) provides an estimate of the population size (*Ne_s_*) as a function of the coalescence rate *λ_n_* (number of coalesced lineages per generation) and the number of lineages (*n*) in the genealogy:

$Ne=\frac{\left(\begin{matrix} n \\ 2 \end{matrix}\right)}{\lambda_{n}}$ (S.Eq.8)

Using time-scaled phylogenetic trees we can estimate the coalescence rate as a function of time *λ_T_* (number of coalesced lineages per time unit), which is connected with the coalescence rate as follows:

$\lambda_{n}=\lambda_{T}T$(S.Eq.9)

Therefore the skyline plot provides an estimate of the product of the coalescence effective population size with the generation time *T.*

$NeT=\frac{\left(\begin{matrix} n \\ 2 \end{matrix}\right)}{\lambda_{T}}$(S.Eq.10)

Kingman and Tavare showed that the coalescence effective population size is connected with the census population size (*N*) and the variance in the reproductive success *σ*^2^ is as follows:

$\frac{Ne}{N}=\frac{1}{\sigma^{2}}$(S.Eq.11)

We follow Felsenstein who showed that this ratio under a Wright-Fisher demography should be 1 and under a Moran demography should be close to 0.5; this has also been supported by simulations for the coalescent effective population size .

The epidemic analogue of the census population size is the number of prevalent cases which we will denote *N*. By combining the skyline plot and the prevalent cases into a ratio we get the following:

$\frac{NeT}{N}=\frac{T}{\sigma^{2}}$(S.Eq.12)

Therefore, if we know *T* we can either estimate the variance in the number of secondary infections per primary infection $\sigma^{2}$ (S.Eq.12) or the proportion of the transmitting individuals of the infected population if *Ne*<<*N* (S.Eq.7).

**2. Phylodynamics estimates of HCV epidemics in Greece.**

*(a)* *PCR-Sequencing*

Regarding the PCR sequencing, best amplification was observed for 1b and 3a, while for 1a and 4a the amplification was less efficient for the E2P7NS2 region (Table S1).

Sequences from different genomic regions in the same patient were concatenated, while sequences from patients in which not all the target regions were amplified were dropped. Our analysis shows that using the tMRCA estimations of E2P7NS2 as a gamma distributed prior to calibrate the NS5B phylodynamic estimations results in increased accuracy. Concatenation of the data resulted in less accurate estimations as previously described . This can be explained by 1) E2P7NS2 containing more molecular clock information, 2) NS5B containing more population structure information due to the increased number of sequenced samples. By concatenating the data and dropping sequences we lose molecular clock information and population structure, resulting in statistically less accurate estimations.

The sequences are deposited in Genbank with the following accession numbers: JN563611-JN563678, FJ538017–FJ538098.

*(b) Phylogenetics – phylodynamics*

Genotype-subtype reference sequences were determined as follows: Genotype 1 (1a: AF009606, AF387806, AF290978, 1b: D50483, AB049093, D85516), Genotype 2 (2a: AF169005, AB047645, D00944, 2b: AB030907, 2c: D50409), Genotype 3 (3a: D28917, D17763, 3b: D49374, 3k: D63821), Genotype 4 (Y11604), Genotype 5 (AF064490, Y13184), Genotype 6 (6a: Y12083, 6b: D84262, 6d:D84263, 6k: D84264, 6h: D84265, 6g: D63822).

Sequence alignment was performed using Clustal-W and was checked manually. We used ModelTest to select the simplest model that fitted the sequence data adequately. Using PAUP , we estimated very large trees (>500 taxa), using neighbor-joining with the Kimura 2-parameter model to determine the distribution of the included samples in the global epidemic. We estimated smaller trees using Tree-Puzzle with the Tamura-Nei model ; rate heterogeneity among sites was modelled using a discrete gamma distribution with four categories of rates.

We performed the main phylodynamic analysis as implemented in BEAST . A Markov chain Monte Carlo (MCMC) was run for each genotype for at least 10,000,000 generations, sampling a tree every 1000 generations. We use the General Time Reversible model of nucleotide substitution with among-site rate heterogeneity modelled using a discrete gamma distribution with four rate categories. The program Tracer (<http://evolve.zoo.ox.ac.uk/software.html?id=tracer>) was used to check for convergence and determine whether appropriate mixing of the MCMC sampler had been achieved in the posterior target distribution (effective sample size>100).

We used relaxed-clock models (uncorrelated log-normal) as implemented in BEAST to determine whether a strict-clock model is appropriate. The strict clock model was then implemented whenever the coefficient of variation (CoV) of the inferred distribution of rates was lower than 0.15, thus indicating consistency with the assumption of a molecular clock. We used the Bayesian skyline model and made no assumptions about the growth and expansion of the epidemic in the population.

We fitted shifted bivariate gamma distributions to the distributions of the time to most recent common ancestor (tMRCA) by calculating the maximum likelihood estimators of the alpha and beta parameters as implemented in STATA 8.0 with the gammafit function . We calculated the shift of the gamma as being equal to the modulus of the minimal value of the estimated distribution of the tMRCA. More details on the phylodynamic approach can be found in a recent publication .

**5. Supplementary References**

1. Wright S (1931) Evolution in Mendelian populations. Genetics 16: 97-159.

2. Wright S (1938) Size of population and breeding structure in relation to evolution. Science 87: 430-431.

3. Kimura M, Crow JF (1963) The measurement of effective population number. Evolution 17: 279-288.

4. Felsenstein J (1971) Inbreeding and variance effective number in populations with overlapping generations. Genetics 68: 581-597.

5. Moran PAP (1962) The Statistical Properties of Evolutionary Theory. Oxford: Clarendon Press.

6. Kingman JFC (1982) On the genealogy of large populations. Journal of Applied Probability 19A: 27-43.

7. Wakeley J, Sargsyan O (2009) Extensions of the coalescent effective population size. Genetics 181: 341-345.

8. Caballero A (1994) Developments in the prediction of effective population size. Heredity 73: 657-679.

9. Creel S (1998) Social organization and effective population sizes in carnivores. Behavioral Ecology and Conservation. Oxford: Oxford University Press. pp. 246-265.

10. Frankham R (1995) Effective population size/adult population size ratios in wildlife: A review. Genet Res Camb 66: 95-107.

11. Frankham R, Ballou JD, Birscoe DA (2002) Introduction to Conservation Genetics. Cambridge: Cambridge University Press.

12. Grant PR, Grant BR (1992) Demography and the genetically effective sizes of two populations of Darwins finches. Ecology 73: 766-784.

13. Nunney L, Campbell KA (1993) Assessing minimum viable population size - demography meets population-Genetics. Trends Ecol Evol 8: 234-239.

14. Nunney L, Elam DR (1994) Estimating the effective population size of conserved populations. Conserv Biol 8: 175-184.

15. Hedgecock D (1994) Does variance in reproductive success limit effective population size of marine organisms? In: Genetics and evolution of aquatic organisms. Beaumont A, editor. London: Chapman and Hall. 122-134 p.

16. Hedgecock D, Chow W, Waples RS (1992) Effective population numbers of shellfish broodstock estimated from temporal variance in allelic frequencies. Aquaculture 88: 215-232.

17. Hedrick P (2005) Large variance in reproductive success and the Ne/N ratio. Evolution 59: 1596-1599.

18. Sargsyan O, Wakeley J (2008) A coalescent process with simultaneous multiple mergers for approximating the gene genealogies of many marine organisms. Theor Popul Biol 74: 104-114.

19. Tavare S, Balding DJ, Griffiths RC, Donnelly P (1997) Inferring coalescence times from DNA sequence data. Genetics 145: 505-518.

20. Frost SD, Volz EM (2010) Viral phylodynamics and the search for an 'effective number of infections'. Philos Trans R Soc Lond B Biol Sci 365: 1879-1890.

21. Magiorkinis G, Magiorkinis E, Paraskevis D, Ho SY, Shapiro B, et al. (2009) The global spread of hepatitis C virus 1a and 1b: a phylodynamic and phylogeographic analysis. PLoS Med 6: e1000198.

22. Thompson JD, Higgins DG, Gibson TJ (1994) CLUSTAL W: improving the sensitivity of progressive multiple sequence alignment through sequence weighting, position-specific gap penalties and weight matrix choice. Nucleic Acids Res 22: 4673-4680.

23. Posada D, Crandall KA (1998) MODELTEST: testing the model of DNA substitution. Bioinformatics 14: 817-818.

24. Swofford D (2003) PAUP*. Phylogenetic Analysis Using Parsimony (*and Other Mehods). Version 4.: Sinauer Associates, Sunderland, Massachusetts.

25. Schmidt HA, Strimmer K, Vingron M, von Haeseler A (2002) TREE-PUZZLE: maximum likelihood phylogenetic analysis using quartets and parallel computing. Bioinformatics 18: 502-504.

26. Tamura K, Nei M (1993) Estimation of the number of nucleotide substitutions in the control region of mitochondrial DNA in humans and chimpanzees. Mol Biol Evol 10: 512-526.

27. Drummond AJ, Rambaut A (2007) BEAST: Bayesian evolutionary analysis by sampling trees. BMC Evol Biol 7: 214.

28. Drummond AJ, Ho SY, Phillips MJ, Rambaut A (2006) Relaxed phylogenetics and dating with confidence. PLoS Biol 4: e88.

29. (2003) Stata Statistical Software: Release 8. In: StataCorp, editor: College Station, TX: StataCorp LP.

30. Lloyd-Smith JO, Schreiber SJ, Kopp PE, Getz WM (2005) Superspreading and the effect of individual variation on disease emergence. Nature 438: 355-359.
